# Regeneration enhancers: a field in development

**DOI:** 10.1152/ajpcell.00403.2022

**Published:** 2022-10-17

**Authors:** Robin E. Harris

**Affiliations:** School of Life Sciences, Arizona State University, Tempe, Arizona

**Keywords:** enhancers, epigenetic, genetics, regeneration, regenerative capacity

## Abstract

The ability to regenerate tissues and organs following damage is not equally distributed across metazoans, and even highly related species can vary considerably in their regenerative capacity. Studies of animals with high regenerative potential have shown that factors expressed during normal development are often reactivated upon damage and required for successful regeneration. As such, regenerative potential may not be dictated by the presence or absence of the necessary genes, but whether such genes are appropriately activated following injury. The identification of damage-responsive enhancers that regulate regenerative gene expression in multiple species and tissues provides possible mechanistic insight into this phenomenon. Enhancers that are reused from developmental programs, and those that are potentially unique to regeneration, have been characterized individually and at a genome-wide scale. A better understanding of the regulatory events that, direct and in some cases limit, regenerative capacity is an important step in developing new methods to manipulate and augment regeneration, particularly in tissues that do not have this ability, including those of humans.

## Graphical abstract

**Figure:**
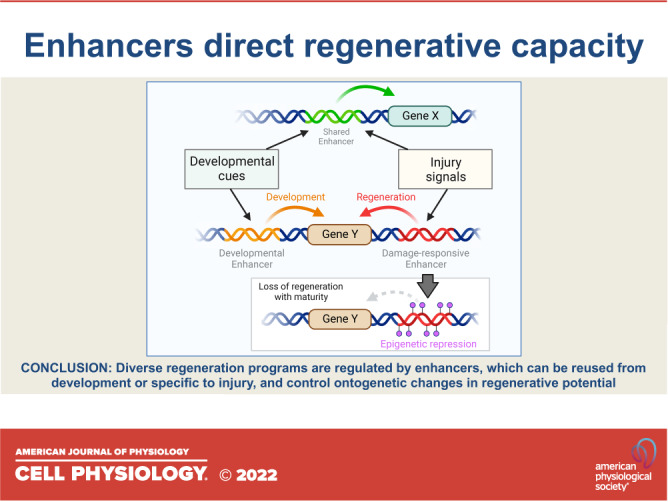

## INTRODUCTION

Regeneration is the ability to regrow tissues and organs that have been damaged or lost through injury or disease (1). Despite likely being an ancestral trait (2), this ability is observed in only a subset of metazoans and can vary considerably, even between highly related species. For example, planarian flatworms such as *Schmidtea mediterranea* can regrow their complete body plan after fragmentation, and even regenerate from a single transplanted stem cell (3). Conversely, other planaria species have reduced or absent regenerative abilities, or abilities restricted to specific systems or structures (3). This variability in regenerative capacity is also observed between highly related species of fish, amphibians, and other vertebrates (2, 4), including mammals (5). Although such variability might suggest that genes specific to regeneration-capable species must exist, few have been identified (6, 7). Rather, it has been consistently shown that factors required for the initial development of an organism also participate in regeneration. Studies show that highly conserved developmental pathways and signaling molecules such as bone morphogenetic proteins (BMPs), FGFs, WNTs, and other growth factors that exist in both regenerative and nonregenerative species are also necessary for tissue regrowth (1, 8, 9). Thus, the potential to regenerate is not solely dependent on the presence of required genes in the genome, but rather the ability to appropriately (re)activate these genes in response to damage. This is supported by the observation that regenerative capacity can vary not only between highly related species but also within a single tissue over time. The ability to regenerate often declines with increasing developmental maturity, a phenomenon seen in diverse species including mice, amphibians, invertebrates, and even humans (10), strongly implying that gene regulation holds the key to regenerative potential.

Although morphological changes in the tissue or systemic changes such as altered hormone availability likely influence regenerative capacity, the dynamics of gene regulation play a crucial role. Recently, enhancers that direct gene expression in response to damage have been identified in several different species (6, 7, 11). These regulatory elements have been given various names, including regeneration signal-response enhancers (RSREs) in *Xenopus* (12), tissue regeneration enhancers elements (TREEs) in zebrafish (13), and damage-responsive elements (DREs) in *Drosophila* (14, 15). For clarity, we will refer to these elements here collectively as regeneration enhancers (REs). Our understanding of how REs function to promote (and in some cases limit) regeneration could provide essential insights into the regulatory mechanisms that underlie regenerative capacity and inform the development of novel methods to augment or stimulate regenerative processes.

## REGENERATION VERSUS DEVELOPMENT

Frequently, genes required for normal development are redeployed during regeneration. A primary example is WNT signaling, which is central to the development of a variety of tissues throughout metazoans, yet is also necessary (and in some cases sufficient) for regeneration in these same organisms, including amphibians, fish, polyps, and flatworms (1, 8, 9, 16). In addition to the reuse of genes themselves, the temporal and spatial expression patterns associated with these genes during development often occur in regeneration. For example, the vertebrate limb is initially formed during embryogenesis by the activity of signaling centers, including the apical ectodermal ridge (AER) and the zone of polarizing activity (ZPA). These regions express genes to coordinate a developmental program that patterns the growing limb along the proximal/distal and anterior/posterior axes (17). Following amputation, a regeneration-specific structure called the apical epithelial cap (AEC) forms, which acts as a comparable signaling center to the AER. Transplantation of AER cells into a severed limb leads to a regenerative response in both chicks and amphibians (18, 19), suggesting the conservation of not only the genetic factors used during development and regeneration but also of the complex regulatory networks that coordinate their expression. Indeed, regulatory elements that are shared between these programs have been identified, including an enhancer known as the ZPA Regulatory Sequence [ZPR, also MFCS1 (20)], which drives Sonic hedgehog (SHH) expression in the ZPA. This enhancer is crucial for normal vertebrate limb patterning during development, whereas CRISPR-mediated deletion of this region in highly regenerative newts inhibits proper limb regrowth following amputation (21). Other examples of developmental enhancers being reused to direct regeneration in cardiac tissue (22, 23) and *Drosophila* imaginal discs (24, 25) have been identified, demonstrating that overlap of gene regulatory mechanisms occurs between development and regeneration.

Despite this overlap, there are certain processes that are unique to each context. Events such as wound closure, immune system activation, and damage-induced changes to cell fate such as dedifferentiation are specific to regeneration (26), and thus necessitate a unique gene expression program. Indeed, there is evidence that regeneration-specific programs take place before the recapitulation of developmental gene expression patterns. Time course experiments have identified unique transcriptomic and epigenetic profiles in the initial stages of regeneration in the axolotl limb, xenopus tail, mouse digit tip, and *Drosophila* imaginal discs (27–30). These programs may reflect the changes necessary to transition from the initial wound healing steps toward an embryonic-like program that redevelops the missing structure. In addition, the events following wound healing do not always exactly recapitulate the developmental program, as deviations specific to the regeneration program have been described in different contexts, including insects (31) and vertebrates (32). The expression of additional factors that regulate cellular identity and positional information that are not present during normal development is perhaps unsurprising, given the distinct anatomical conditions of regenerating tissue versus embryogenesis, (the growth of a nascent limb from a mature shoulder, for example), and the presence of mature gene expression programs. Moreover, the signaling events of wound healing have been shown to influence subsequent cell fate regulation, thus requiring unique nondevelopmental mechanisms to ensure correct repatterning during regeneration (31). However, how specific the initial wound response program and the subsequent events that sustain regrowth are to regeneration, and their overlap with developmental programs, remain to be fully explored.

## REGENERATION ENHANCERS

**Figure 1.**
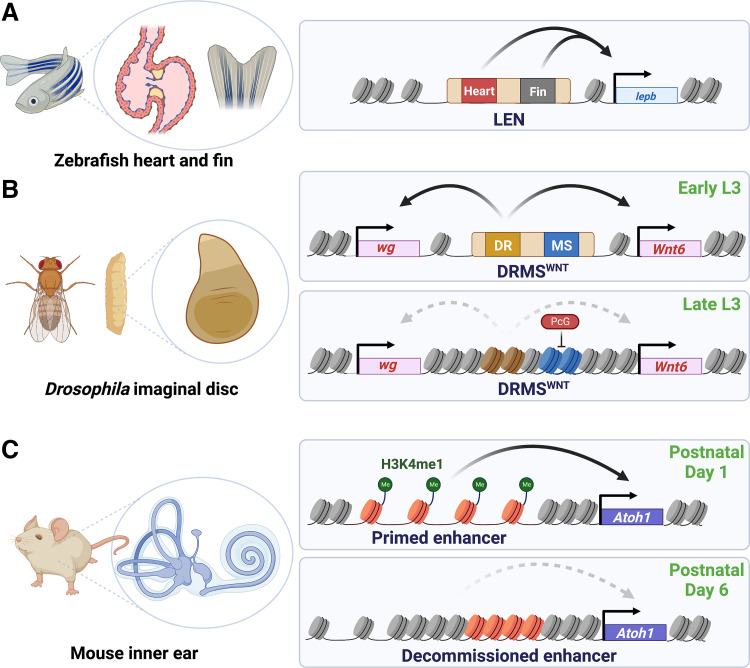
Characterized regeneration enhancers (REs) and how they regulate gene expression in different tissue contexts of fish, flies, and mice. *A*: the *lepb*-linked enhancer (LEN) with separable tissue-specific modules that drive regenerative *lepB* expression in response to cardiac or appendage (fin) injury (13). *B*: the damage-responsive maturity-silenced enhancer of the WNT locus (DRMS^WNT^, also BRV118) in, which activates *wg* and *Wnt6* in response to damage in early third instar (L3) wing imaginal discs. Like the LEN, DRMS^WNT^ also has modular elements, but in this RE they regulate maturity-dependent activity in a single tissue rather than organ-specific behavior. The damage-responsive (DR) region mediates JNK-dependent expression of the flanking WNT genes, whereas the maturity-silencing region (MS) nucleates localized epigenetic silencing of the enhancer as the disc develops from early to late L3, coinciding with its loss of regenerative potential. Silencing is dependent on Polycomb group (PcG) proteins, manipulation of which can improve regenerative capacity (25). *C*: REs in the inner ear that regulate the transdetermination of support cells into auditory hair cells in response to their loss in early neonatal mice. Multiple REs associated with hair cell-specific genes, such as *Atoh1*, are silenced but primed in support cells through the addition of H3K27me3 and H3K4me1 marks (34). Within a week of maturation, the H3K4me1 marks are removed to decommission REs and curtail regenerative gene expression. [Image created with BioRender.com and published with permission.]

A single gene used during both regeneration and development is activated by different initiating events; damage-induced signals versus programmed developmental cues. Until recently it was not clear how this might be achieved, but the discovery of regeneration-specific regulatory elements that drive gene expression following injury has provided an answer. The teleost zebrafish (*Danio rerio*) has a remarkable regenerative capacity of the heart, fins, brain, spinal cord, and other tissues, which is maintained throughout their life (33). Upon damage to fins and cardiac tissue, the *leptin b* (*lepb*) gene, which encodes a homolog to mammalian *leptin*, is strongly activated at the site of injury (13). Histone acetylation marks were used to detect active enhancer regions around this gene, which identified a short DNA sequence named the *lepb*-linked enhancer (*LEN*, Fig. 1*A*). Transgenic reporters of this DNA sequence were active during regeneration but not development (13), and deletion analysis showed it was necessary for *lepb* upregulation following fin amputation (35). The LEN enhancer was also found to be modular, exhibiting separable regions that can direct expression in either the fin or the heart upon damage (13). Thus, the expression of a single proregeneration gene can be activated by different elements within this RE to provide a tissue-specific regenerative response. Moreover, when engineered into mice and paired with a murine promoter, this RE showed damage-induced activity in both amputated digit tips and the heart, illustrating the conservation of this mechanism (13). Using histone replacement profiling, ChIP-seq and chromatin accessibility profiling (ATAC-seq) to identify active enhancers, it was further shown that damage induces thousands of accessibility changes that potentially represent REs like the LEN enhancer, which have activity during either cardiac or fin regeneration but not during the development of these same structures (35, 36). Thus, in zebrafish, a regeneration program comprising many thousands of genes may be activated and/or coordinated by these discrete regulatory regions.

The specificity of this zebrafish regeneration program was recently tested by comparing it with that of a related teleost, the African killifish (*Nothobranchius furzeri*; 37). Genome-wide analyses identified many species-specific transcriptomic and chromatin-level changes induced upon fin amputation, whereas a smaller number of gene expression changes common to both species were also detected. The genes in this common pool were associated with a collection of REs, which together may represent a core regeneration response program that has been maintained in both teleost species despite 230 million years of evolutionary distance (37). The RE of one of these factors, *inhba*, was also found to be conserved in humans, whereas a direct comparison of how the killifish, zebrafish, and human *inhba* REs behave following damage suggest that, despite persisting in each organism, the function of this region has diversified in nonregenerating humans versus regeneration-capable teleosts (37).

The experiments in zebrafish demonstrate that REs are potentially widespread across in the genome and regulate multiple genes of the regeneration program. However, the question remains how the activity of these regions is coordinated upon damage. This question was explored in the highly regenerative acoel worm *Hofstenia miamia*, which can regenerate its entire body plan following physical fragmentation (38). Combining ATAC-seq and RNA-seq with a new genome annotation for the species, it was shown that many thousands of potential REs likely exist. Transcription factor binding motif predictions identified a single factor, early growth response (Egr), that dynamically regulates many of these regions (38). This was confirmed by functional knockdown of *Egr*, which revealed the target genes that form a regulatory network required for whole body regeneration. Together these data show that a complex regeneration program consisting of multiple interacting genes can be launched and controlled by a single “master regulator” acting via discrete REs (38).

The genome-wide identification of REs has also been pursued in other invertebrates, including *Drosophila* (14, 15). The imaginal discs of *Drosophila* are larval epithelial tissues that are the precursors to adult structures, such as the legs and wings. The wing imaginal disc has been studied for decades to understand diverse aspects of developmental growth and has since become a leading model to explore the fundamental genetic events underlying regeneration (39). Pairing genetic ablation with chromatin profiling, the gene expression profile and changes in regulatory element landscape have been mapped at progressive timepoints in regenerating discs (14), and at different developmental stages (15). As in Zebrafish and the acoel worm, a defined set of chromatin accessibility changes were found to occur at hundreds of genomic sites, which were correlated with characteristic changes in histone modifications to suggest active REs (14, 15). These regions could be categorized into preexisting enhancers that become more accessible upon damage and novel enhancers that become accessible strictly following injury. Many of the latter appeared to overlap with regions identified as developmental enhancers that are active during patterning of other imaginal discs, or at different developmental stages, suggesting that this group of enhancers consists of those reused from development, as well as those specific to regeneration (14). A similar pattern of developmental enhancer reuse was found when this same chromatin profile analysis was applied to zebrafish cardiac regeneration (14). Thus, the reuse of developmental enhancers in regeneration may not be limited to a few individual examples, such as the ZPR used in both limb formation and regrowth, but might be a conserved aspect of regeneration programs. Performing comparisons between genome-wide chromatin changes that occur during regeneration and in distinct developmental programs (e.g., embryonic vs. juvenile, or in individual tissues) in different species may provide insight into how REs have evolved to either be maintained, repurposed, or lost between species that vary in regenerative ability.

Beyond these examples, there is evidence that REs exist in other organisms including mice (40) and frogs (12), some of which may even be conserved in humans. For instance, kidney regeneration is highly variable between species, but *Xenopus* can completely regenerate damaged renal tubule structures (41). Investigation of *lxh1*, a gene involved in kidney formation, showed that it is also required for regeneration following partial nephrectomy in the frog (12). To identify potential regulatory elements relevant to regeneration, researchers used the degree of similarity of noncoding sequences surrounding the *lxh1* gene in frogs, fish, and humans to detect conserved genomic regions, and located several regions that can drive damage-induced activity of *lxh1* in reporter assays. The Arid3a transcription factor was found to bind at these REs, acting to reduce histone methylation and thus promote *lxh1* expression in response to injury (12). This assay illustrates that REs for a particular proregeneration factor can maintain conservation between distantly related vertebrates and that this property can be used to identify such enhancers, in addition to chromatin profiling. A similar methodology was also used to detect enhancers that direct *Bmp5* expression in developing mice, providing a basis to identify regulatory regions that act specifically to drive reparative Bmp5 following bone fractures and soft tissue injuries to the lung and skin (40). As mammals have comparatively poor regenerative ability, these enhancers were considered in the context of wound healing rather than regeneration, supporting the idea that damage-responsive enhancers used in regenerative species may have diversified during evolution to regulate wound healing processes in nonregenerative animals (37). Additional repair-related enhancers have been identified through conservation analysis or chromatin profiling in mammalian models of muscle injury (42), cardiac repair (22, 23), and in the formation of repair Schwann cells following peripheral nerve damage (43). There is even evidence that such regulatory elements function in models of human epithelial injury (44).

Importantly, in many of the enhancers identified across diverse species, there is often an enrichment or demonstrated requirement for binding sites of the AP-1 transcription factor (Jun/Fos), an effector of the JNK pathway (12–14, 25, 35, 37, 38, 40, 42, 43). This regulatory signature, alongside other common features such as dynamic changes in epigenetic accessibility and predictable histone modifications, may reveal additional conserved features that could be used to better predict putative REs going forward.

## ENHANCERS CONTRIBUTING TO ONTOGENETIC CHANGES IN REGENERATIVE CAPACITY

Although some organisms maintain the ability to regenerate throughout their lives, regenerative capacity often declines as development proceeds (10), providing a valuable opportunity to identify and characterize the genes and regulatory elements that permit damage-induced tissue regrowth. In most contexts, it is currently unknown whether REs contribute to ontogenetic changes in regeneration. However, several examples of enhancers that become inactive to limit regenerative gene expression have been identified. One of the best-defined examples is that of the damage-responsive, maturity-silenced (DRMS) enhancer of the WNT locus in *Drosophila* (15, 24, 25, Fig. 1*B*). The *Drosophila Wnt1* ortholog *wingless* (*wg*) and the *Wnt6* genes are both expressed in response to damage in the larval wing imaginal disc (24, 25). However, this expression becomes limited as larvae mature through the third larval instar (L3), coinciding with a loss of this tissue’s regenerative ability. A regulatory region [DRMS^WNT^, also BRV118 (45)] was identified between the WNT genes, which is directly activated by AP-1 upon injury (25). Deletion of this 3 kb genomic region was found to limit damage-induced WNT expression and impair regeneration, demonstrating its necessity for tissue repair (24, 25). Analyses using reporter transgenes alongside targeted deletions of different elements within this region showed that discrete cis-regulatory modules (CRMs) act together to promote developmental WNT expression required for the initial formation of the wing pouch in the disc before L3 (24), and to mediate injury-induced signals to activate damage-responsive WNT expression during regeneration in subsequent L3 (24, 25). Importantly, this damage-induced activity occurs strongly in blastema cells of early L3 discs but becomes progressively limited as the discs mature, concurrent with the loss of regenerative ability. Dissection of the DRMS^WNT^ enhancer and analyses at both early and late L3 stages revealed that a distinct 1 kb region downstream of the characterized developmental and damage-activated CRMs contains elements that mediate increasing epigenetic silencing dependent on Polycomb group (PcG) proteins (25). By analyzing the DNA sequence of this RE, an additional DRMS was identified at another gene with a prominent role in regeneration, *matrix metalloproteinase 1* (*Mmp1*; 15). The DRMS^Mmp1^ enhancer was also found to be modular, with distinct activating and silencing CRMs. Evaluating the chromatin of each enhancer showed that both regions normally increase in accessibility upon damage in regeneration-competent early L3 discs but fail to do so in late L3 discs. This assay also identified dozens of other regions across the genome whose behavior mirrored that of the DRMS^WNT^ and DRMS^Mmp1^ enhancers, suggesting that multiple genes comprising a regeneration program might be coordinately regulated by this mechanism, which act collectively to dictate the regenerative potential of the tissue as it develops (15).

The loss of regenerative potential via epigenetic silencing of REs is not limited to invertebrates. Within the mammalian inner ear, mechanosensitive hair cells in the cochlea translate auditory stimuli into nerve signals to the brain. Loss of these cells due to injury or aging leads to hearing impairment, a cause of deafness in humans. Nonmammalian vertebrates have a robust capacity to replace these cells through the transdifferentiation of support cells into hair cells (46). By contrast, adult mice are unable to do so. However, there is a brief period at the beginning of neonatal life in which these support cells are able to replace lost hair cells (47). This process is dependent on their ability to launch a hair cell differentiation program, which is normally silenced but remains primed via a histone modification (H3K4me1) at enhancers that regulate hair cell gene expression (34; Fig. 1*C*). The loss of hair cells triggers the activation of these enhancers and thus the expression of genes such as *Atoh1*, leading to transdifferentiation into hair cells. However, as the inner ear matures in the first week of life, this primed state is lost and the enhancers are inactivated, limiting the regenerative ability of the tissue with age. Unlike the cochlea, this enhancer decommissioning does not occur in the supporting cells of the inner ear vestibular system, correlating with the limited but persistent regenerative potential of support/hair cells in this system (48).

Although we have an incomplete understanding of the contribution that REs make to the loss of regenerative capacity, there is evidence this regulatory mechanism may also occur in other vertebrates. For example, the loss of regeneration of *Xenopus* limbs correlates with increasing DNA methylation at the ZPR (MFCS1) enhancer that drives regenerative SHH expression, suggesting a relationship between the epigenetic status of an RE and regenerative potential (49). In the future, it will be important to examine if the activity of such regulatory elements coincides with changes in regenerative capacity in other species, and test whether manipulations of these REs could prolong regenerative capacity.

## IMPROVING REGENERATIVE CAPACITY BY MANIPULATING ENHANCER FUNCTION

Promoting regeneration is a major therapeutic goal that may be realized through a better understanding of how regulatory elements contribute to regenerative capacity. Two different methods have been developed that improve regeneration by manipulating enhancers in both zebrafish (13) and *Drosophila* (15, 25). One approach harnesses the enhancer regions themselves to drive ectopic expression of proregeneration genes. Positioning the LEN enhancer upstream of a regeneration-promoting gene, *fgf20a*, rescues fin regeneration defects in *fgf20a* mutant zebrafish (13). Moreover, using the LEN enhancer to drive the known cardiomyocyte mitogen *nrg1* significantly increases regenerative proliferation in damaged heart tissue. Importantly, the ectopic mitogen levels decline after cardiac regeneration is complete, while *fgf20a* expression does not produce any uncontrolled fin growth, suggesting the activity of these factors is restricted to the period of regeneration. A similar approach was used in *Drosophila* using the damage-responsive module of the DRMS^WNT^ enhancer to drive expression of the progrowth gene *myc* (25). As with the LEN enhancer, gene expression was activated upon damage, spatially limited to blastema cells, and persisted only until regeneration was completed. These experiments confirm that known REs can be used to target spatially and temporally limited expression of mitogens to augment regeneration.

A second approach to improve regeneration in *Drosophila* focused on altering the activity of enhancer regions themselves (15). It is known that mutation or knockdown of different chromatin modifiers, including PcG genes, can promote or constrain regeneration of imaginal discs (15, 50). Knowing that PcG proteins silence DRMS activity in late L3 discs, which potentially curtails regenerative capacity, a targeted RNAi screen to knockdown different PcG genes was performed. The limited knockdown of the PRC2 gene extra sex combs (*esc*, *Eed* in mammals) was found to increase the activity of multiple REs following damage and significantly improve regeneration (15). Increasing the repair potential of a tissue by epigenetic manipulation was similarly achieved in mice. Blocking histone demethylation in support cells of the inner ear prolonged the primed enhancer state that permits these cells to transdifferentiate into hair cells (34), demonstrating that altering the epigenetic status of REs can also be used to augment regenerative capacity in mammals.

The examples outlined here illustrate that although researchers have identified many factors that participate in regeneration, the mechanisms by which they are activated and coordinated are only now starting to be characterized. Pursuing a full understanding of REs will provide valuable insights into the fundamental mechanisms that underlie regeneration, which is essential for ultimately developing novel therapeutic treatments relating to improving regeneration in vivo.

## GRANTS

This work was supported in part by the Eunice Kennedy Shriver National Institute of Child Health and Human Development Grant R21HD102765 and the National Institute of General Medical Sciences Grant R01GM147615.

## DISCLOSURES

No conflicts of interest, financial or otherwise, are declared by the authors.

## AUTHOR CONTRIBUTIONS

R.E.H. drafted manuscript; edited and revised manuscript; approved final version of manuscript.
